# A Forensic Genomics Approach for the Identification of Sister Marija Crucifiksa Kozulić

**DOI:** 10.3390/genes11080938

**Published:** 2020-08-14

**Authors:** Charla Marshall, Kimberly Sturk-Andreaggi, Erin M. Gorden, Jennifer Daniels-Higginbotham, Sidney Gaston Sanchez, Željana Bašić, Ivana Kružić, Šimun Anđelinović, Alan Bosnar, Miran Čoklo, Anja Petaros, Timothy P. McMahon, Dragan Primorac, Mitchell M. Holland

**Affiliations:** 1 Armed Forces Medical Examiner System (AFMES), Dover Air Force Base, Dover, DE 19902, USA; kimberly.s.andreaggi.ctr@mail.mil (K.S.-A.); egorden@gmail.com (E.M.G.); jennifer.l.higginbotham3.ctr@mail.mil (J.D.-H.); sidney.a.gaston-sanchez.ctr@mail.mil (S.G.S.); Timothy.p.mcmahon10.civ@mail.mil (T.P.M.); 2 SNA International, Contractor Supporting the AFMES, Alexandria, VA 22314, USA; 3 Department of Biochemistry & Molecular Biology, Forensic Science Program, The Pennsylvania State University, University Park, PA 16802, USA; draganprimorac2@gmail.com; 4 Department of Forensic Sciences, University of Split, 21000 Split, Croatia; zbasic@unist.hr (Ž.B.); ivana.kruzic@unist.hr (I.K.); 5 Medical School, University of Split, 21000 Split, Croatia; simun.andjelinovic@unist.hr; 6 Clinical Department for Pathology, Legal Medicine and Cytology, Clinical Hospital Center Split, 21000 Split, Croatia; 7 Department of Forensic Medicine and Criminalistics, University of Rijeka School of Medicine, 51000 Rijeka, Croatia; alan.bosnar@medri.uniri.hr; 8 Institute for Anthropological Research, Center for Applied Bioanthropology, 10000 Zagreb, Croatia; miran.coklo@inantro.hr; 9 National Board of Forensic Medicine, Department of Forensic Medicine, 58758 Linköping, Sweden; anja.petaros@yahoo.com; 10 St. Catherine Specialty Hospital, 49210 Zabok/10000 Zagreb, Croatia; 11 School of Medicine, University of Osijek, 31000 Osijek, Croatia; 12 Faculty of Dental Medicine and Health, University of Osijek, 31000 Osijek, Croatia; 13 The Henry C. Lee College of Criminal Justice and Forensic Sciences, University of New Haven, New Haven, CT 06516, USA; 14 School of Medicine, University of Rijeka, 51000 Rijeka, Croatia; 15 Medical School REGIOMED, 96450 Coburg, Germany

**Keywords:** forensic genomics, human identification, historical remains, beatification, mitogenome, short tandem repeat (STR), single-nucleotide polymorphism (SNP), kinship inference, massively parallel sequencing (MPS), next-generation sequencing (NGS)

## Abstract

Sister Marija Krucifiksa Kozulić (1852–1922) was a Croatian nun who is in consideration for beatification by the Vatican, which is facilitated by the identification of her 20th-century remains. Sister Marija was buried in a tomb in Rijeka, Croatia, along with other nuns including her biological sister, Tereza Kozulić (1861–1933). When the remains were exhumed in 2011, they were found in a deteriorated state and commingled with several other sets of remains. Thus, mitochondrial genome sequencing of the long bones was performed to sort the remains by mitochondrial haplotype. Two similar but unique haplotypes belonging to haplogroup H1bu were identified, and samples from these bones were subjected to autosomal short tandem repeat (STR) and single nucleotide polymorphism (SNP) sequencing. Although only partial profiles were obtained, the data were sufficient for kinship analysis with the profile of a paternal niece of Sister Marija (Fides Kozulić). The data indicate that it is 574,195-fold more likely that the two sets of skeletal remains represent 2nd-degree relatives of Fides than sisters who are unrelated to Fides. Although it is impossible to discern which set of remains belongs to Marija and which belongs to Tereza, forensic genomics methods have enabled identification of the sisters.

## 1. Introduction

**Figure 1:**
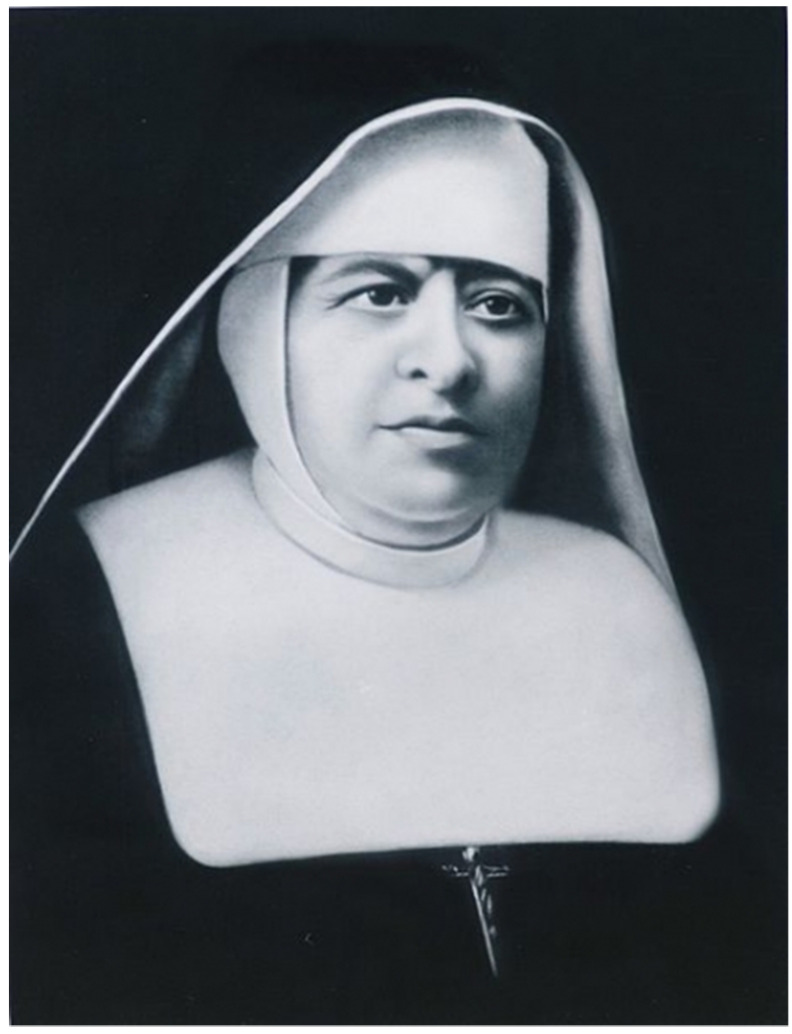
Reproduced photograph of Sister Marija Krucifiksa Kozulić (1852–1922) [1].

**Figure 2:**
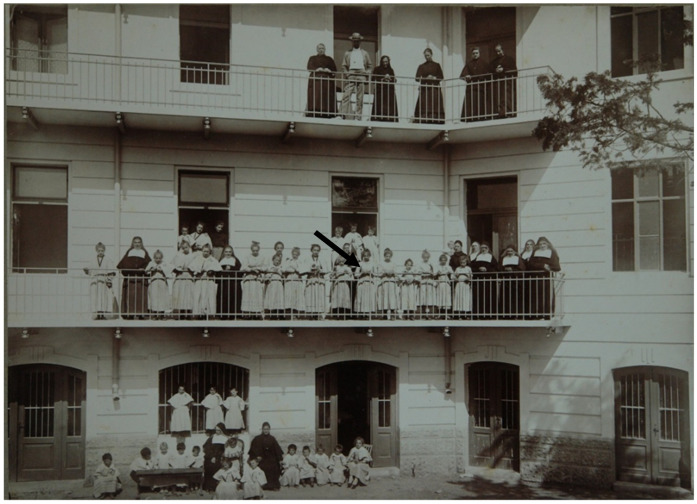
Sister Marija (first floor, holding a Jesus stature, in the center, arrow pointing at Sister Marija) with her brother (second floor), sister Tereza (first floor, holding a baby in her arms, closest nun to Sister Marija on the right), and monastery nuns and orphans.

Sister Marija Krucifiksa Kozulić (1852–1922) was a pious and generous nun from Croatia who dedicated her life to helping the poor and less fortunate (Figure 1). During World War I, Sister Marija lived on the island of Krk where she ran an orphanage (Figure 2). After the war, she moved to Rijeka where she died of stroke on 29 September 1922, at the age of 70. She is currently in consideration for beatification by the Vatican, which is facilitated by the identification of her 20th-century remains. Upon her death, Sister Marija was buried in a tomb belonging to the Society of Sisters of the Sacred Heart of Jesus in Rijeka, along with other nuns including her biological sister, Tereza Kozulić (1861–1933). A total of 52 individuals were known to be buried in the tomb based on historical records. On the 20th of February 2011, these remains were exhumed for the purposes of Sister Marija’s identification. 

The tomb consisted of three layers of burials (Figure S1). Thirty-five individuals were found buried in tin coffins, wooden coffins, and wooden boxes; these persons were identified by name during the anthropological assessment. The remaining sets of skeletal remains were discovered in seven plastic bags. The transfer of bones from deteriorated coffins to these plastic bags was most likely done in 2006, when the tomb was reorganized to make room for newly buried remains. Except for one plastic bag that contained the remains of a single individual, the remaining six plastic bags contained a commingled assemblage of incomplete skeletal remains. The plastic bags created a humid environment that propagated greater degradation of the bones and the development of mold in some areas. Thus, the remains were exposed to different taphonomic conditions that complicated the re-articulation process. The minimum number of individuals in the plastic bags, based on the numbers of left femora and right tibiae, was nine. However, the plastic bags should have contained the remains of seventeen persons, based on the total number of known burials in the tomb. Therefore, the possibility that eight persons were missing from the exhumations could not be ruled out. 

In an attempt to identify the remains of Sister Marija, DNA analysis was performed on the unidentified skeletal remains from the tomb. First, the femoral bones (and two humeri) were subjected to mitochondrial (mt) DNA analysis in order to screen for maternal relatives, as the only two known to be buried in the tomb were Sister Marija and Sister Tereza. Once the remains of putative maternal relatives were found, autosomal DNA testing was performed to confirm the suspected sibling relationship. This two-step approach to DNA testing of the historical remains allowed for an initial assessment of DNA quality through the mtDNA analysis, and it maximized the cost effectiveness of the study by limiting the autosomal DNA testing and sequencing runs required. Finally, the identity of the sisters was assessed through kinship inference to a DNA sample from the only available relative of Sister Marija, Fides Kozulić, the paternal niece of Marija and Tereza. This kinship assessment was necessary because direct DNA references from Sister Marija, such as hair from a brush or a personal clothing item, were not available. This study summarizes the results of the DNA testing performed on the remains of the tomb for the Sisters of the Sacred Heart, and the DNA evidence that can be used in the identification of Sisters Marija and Tereza.

## 2. Materials and Methods 

### 2.1. Sample Selection

#### 2.1.1. Skeletal Samples 

Following exhumation, the samples were taken to the Department of Legal Medicine and Criminalistics, Rijeka University. The bones were processed following Scientific Working Group of Forensic Anthropology protocols. The bones were cleaned with water (no detergents or chemicals) and a soft brush. The remains were in poor condition so porous bones were not exposed to water but were cleaned with a soft brush. The remains cleaned with water were left to dry at ambient temperature. The long bones were transferred to the Clinical Hospital Center, Split, where they were measured and prepared for sampling. Samples of long bones were washed using tap water and a soft brush, and after the cleansing, they were washed with deionized water and left to air dry. After drying, the samples were cut using a dental saw (KaVo Elektrotechnisches Werk, Vertriebsgesellschaft GmbH, Leutkirch, Germany). An approximately 5–15 g portion of each of the fourteen long bone samples was taken for DNA analysis (Table S1). 

#### 2.1.2. DNA Reference Sample 

For identification purposes, there were no direct reference samples from Sister Marija, such as a brush containing hairs, blood, or other sources of DNA. Her breviary and rosaries exist, but these have been used by the other monastery nuns since Marija’s death, and through time have been cleaned. Only one living relative was found, Fides Kozulić, who is a daughter of Marija and Tereza’s brother. Fides provided a buccal sample and a blood sample for the analysis prior to her death in 2013. As a paternal relative, Fides would not be a suitable reference for mtDNA that is inherited maternally. Therefore, only autosomal DNA analysis was performed on the samples from Fides Kozulić.

### 2.2. DNA Extraction 

#### 2.2.1. Skeletal Samples 

Approximately 500 mg of bone powder was digested at 56 °C overnight in a 0.5 M EDTA (Sigma-Aldrich; St. Louis, MO, USA) and 1% w/v n-laurylsarcosine (Sigma Aldrich) buffer with 10 mg of proteinase K. DNA was purified either by organic extraction [2] or by centrifugal filtration using Ultra-4 centricons (Millipore Sigma; Burlington, MA, USA) followed by MinElute purification (QIAGEN; Hilden, Germany) [3]. The organic DNA extracts underwent DNA repair using the NEBNext FFPE DNA Repair Mix (New England Biolabs; Ipswich, MA, USA) followed by MinElute purification (QIAGEN) [4]. 

Two or more independent DNA extracts were produced from each sample for replication of results, including replicate mtDNA sequence analysis at both The Armed Forces Medical Examiner System—Armed Forces DNA Identification Laboratory (AFMES-AFDIL) and The Pennsylvania State University. A reagent blank was included in each extraction set. 

#### 2.2.2. Buccal Swab

A buccal swab was collected from Fides Kozulić in 2011 as a DNA reference of Sister Marija Kozulić, her paternal aunt. The buccal swab was cut into two halves using a sterile blade and placed in 1.7 mL tubes. DNA extraction was performed independently on each half of the swab on separate occasions using the QIAamp DNA Investigator kit (QIAGEN) following the manufacturer’s recommendations. 

### 2.3. DNA Quantitation 

DNA quantitation was performed as a quality control check using one of three assays. The Plexor HY real-time PCR assay (Promega Corporation; Madison, WI, USA) was used for nuclear DNA quantitation. The Qubit dsDNA High Sensitivity (HS) assay (Thermo Fisher Scientific; Waltham, MA, USA) was used to determine total genomic DNA content. An mtqPCR assay [5] was performed to assess mtDNA content.

### 2.4. Mitochondrial Genome Sequencing 

Illumina libraries were prepared using the KAPA Hyper Prep kit (Roche Sequencing; Pleasanton, CA, USA). A hybridization capture method was used to enrich for the entire mitochondrial genome (mitogenome) and capture was performed with a custom myBaits kit (Arbor Biosciences; Ann Arbor, MI, USA) described in [3]. The capture product was amplified with KAPA HiFi ReadyMix PCR Kit (Roche Sequencing) and the KAPA Library Amp Primer Mix (Roche Sequencing), targeting the universal Illumina primers (P5: 5′ AAT GAT ACG GCG ACC ACC GA 3′; P7: 5′ CAA GCA GAA GAC GGC ATA CGA 3′). PCR products were purified with AMPure XP PCR bead purification (Beckman Coulter; Indianapolis, IN, USA). Purified products were quantified on a 2100 Bioanalyzer (Agilent Technologies, Inc.; Santa Clara, CA, USA) with the Agilent High Sensitivity DNA kit (Agilent Technologies, Inc.).

Samples were pooled and normalized to 4 nM. The pooled library was spiked with 5% PhiX Sequencing Control v3 (Illumina; San Diego, CA, USA) and sequenced on a MiSeq or MiSeq FGx Forensic Genomic System (Verogen; San Diego, CA, USA) in the Research Use Only mode using a MiSeq v2-300 cycle reagent kit (Illumina) for 150 × 2 paired-end sequencing.

### 2.5. Mitogenome Sequence Analysis 

MiSeq Reporter (Illumina) was used to demultiplex the sequence data, remove barcodes, trim adapters, and filter low quality reads. The FASTQ files generated by MiSeq Reporter were analyzed in CLC Genomics Workbench version 7.5.1 (QIAGEN) following the methods detailed in [3]. Briefly, paired-end sequences were mapped to the rCRS [6] using stringent alignment parameters to preclude the mapping of off-target reads. Mapped duplicates were removed, and variants above 5% frequency were reported in each haplotype. The AFDIL-QIAGEN mtDNA Expert (AQME) tool [7] was used to analyze length variants, conform the variant profile to forensic nomenclature, and estimate the mitochondrial haplogroup. 

Data analysis was also performed using the GeneMarker HTS software (SoftGenetics; State College, PA, USA) version 1.2.2 [8]. FASTQ files generated from the MiSeq Reporter software (Illumina) were aligned to the rCRS. A custom motif file was used to ensure phylogenetically correct calls. The minimum read depth at each nucleotide and minor variants were 10X. Additional parameters for variant calling were also applied including an allele score difference of ≤10, a single-nucleotide polymorphism (SNP) balance ratio of ≤2.5, and an insertion/deletion balance ratio of ≤5.0. The analytical threshold was 1%. However, due to the nature of the challenged samples, the reporting threshold for heteroplasmy was 5%.

### 2.6. Autosomal STR and Identity SNP Sequencing 

Based on the results of the mtDNA sequencing, autosomal DNA data were generated for bone samples with the same mtDNA haplotypes and the buccal swab from Fides Kozulić. An additional bone sample with a different mtDNA haplotype (i.e., presumed unrelated) was included as a “control” to test for adventitious relatedness in this unique historical context. Three associated extraction blanks, three amplification blanks, and a positive control (2800M) were also processed simultaneously. Autosomal short tandem repeat (STR) and SNP targets were PCR amplified using the Applied Biosystems Precision ID Identity SNP and GlobalFiler NGS STR panels (Thermo Fisher Scientific). The Identity SNP panel includes 90 unlinked autosomal SNPs and 34 upper Y-clade SNPs. The Globalfiler NGS panel v1 includes 29 autosomal STRs as well as amelogenin (X-Y paralog) and 2 Y-chromosomal markers. PCR product was purified using a 1.8X Agencourt AMPure XP purification reaction and DNA was eluted in Tris-EDTA [10 mM Tris, (pH 7.5) 0.1 mM EDTA] prior to library preparation for Illumina sequencing. The KAPA Hyper Prep kit (Roche Sequencing) was used to prepare dual indexed Illumina libraries with 12 cycles of PCR amplification. Amplified libraries were quantified using the Agilent DNA 7500 Kit (Agilent Technologies, Inc.) on the 2100 BioAnalyzer instrument. Libraries were normalized and pooled, then spiked with denatured PhiX Sequencing Control v3 at either 2.5% or 5% v/v concentration. Paired-end sequencing was performed on a MiSeq FGx Forensic Genomic System in the Research Use Only mode using a MiSeq v3 600-cycle reagent kit (Illumina). To improve coverage of the SNP targets, some of the libraries were resequenced on an Illumina NextSeq 550 using a Mid-Output 300 cycle kit (Illumina) loaded at 1.2 pM with 5% PhiX for single-end sequencing.

### 2.7. Autosomal STR and Identity SNP Data Analysis 

FASTQ files produced by MiSeq Reporter from the Precision ID STR and SNP Illumina sequencing were analyzed in the Parabon Fx Forensic Analysis Platform (Parabon Nanolabs; Reston, VA, USA). The STR data were analyzed by sequence, but only the length-based alleles were reported. The reason for this approach was the improved resolution of stutter products. However, since sequence-based allele frequency data are lacking, downstream analyses required length-based alleles. STR and SNP data were analyzed using a minimum read depth of 10 for each locus, and heterozygous loci were reported when the minor allele exceeded 20% frequency.

Consensus STR and SNP profiles were created for each sample by calling only the alleles that were independently replicated in two or more DNA extracts [9,10]. If only one allele was replicated, the locus was reported as a homozygote. This approach allowed for loci to be reported regardless of heterozygote dropout in order to maximize the number of authentic alleles contained in the final DNA profile. Amelogenin and the presence/absence of the Y-chromosomal markers were used to confirm the sex of the samples tested.

### 2.8. Kinship Inference 

Kinship analysis was performed in Familias 3 [11,12]. Allele frequencies from the 1000 Genomes European population were used for 89 of the 90 autosomal SNPs [13,14]. One SNP (rs938283) had no allele frequencies available in the 1000 Genomes data, and thus allele frequencies from the ALFA European dataset were used [15]. STR allele frequencies from the NIST U.S. Caucasian (European ancestry) population data were employed [16,17]. The two sets of allele frequencies were combined to encompass all 90 SNP and 29 STR autosomal markers included in the Precision ID Identity SNP and Globalfiler NGS panels, respectively. The consensus profiles for bone samples and the buccal swab (Fides Kozulić) were imported into Familias. The unrelated “control” sample was included in these analyses to test for adventitious relatedness. Such a false-positive relationship could potentially originate from sampling bias due to the age, location, and time period of the bones sampled from this unique historical case, combined with the allele frequency data available for the kinship analysis (individuals of contemporary European ancestry). The bias could be further exacerbated by allelic dropout from DNA degradation. To account for this, the control bone sample from a set of remains with a different mtDNA haplotype was included in the kinship analysis. A blind search was performed in order to test for any genetic relationships between the tested samples. The likelihood ratios (LRs) for parent–child, full siblings, 2nd-degree (e.g., half siblings or avuncular), and 3rd-degree (e.g., cousins) relationships versus unrelated were calculated [18]. Using equal priors, a posterior probability was obtained based on the calculated LRs to assess the most probable degree of relatedness (if related) between the tested samples. Pedigrees were created based on the developed hypotheses from the mtDNA testing, as the only two maternal relatives in the tomb were Sister Marija and Tereza, and the likelihoods of each hypothesis were compared.

## 3. Results

### 3.1. Mitogenome Sequencing

Twelve of the 14 skeletal samples produced complete mitogenome sequence data from two or more independent DNA extracts (Table 1). Samples 45 and 46 failed to yield reproducible sequence data. Sample 45 produced mixed sequence data from one DNA extract (45.1) and a partial profile from the second DNA extract (45.2). Sample 46 produced very few reads that mapped to the rCRS (<250 per DNA extract). It is of note that neither of these two femoral bone samples could be sided during the anthropological analysis, indicating poor gross preservation of the bone that coincided with the poor DNA quality. Sequence data from the 12 samples varied in terms of read count as well as the percentage of reads that mapped to the rCRS. Average read depth ranged from 47X to 17,251X, and complete sequence ranges (16,569 bp with at least 10X read depth) were obtained for each replicate of the 12 successful samples. None of the control blanks (extraction blanks and negative library controls) showed evidence of contamination following the guidelines described in [3] (Table S2).

**Figure 3:**
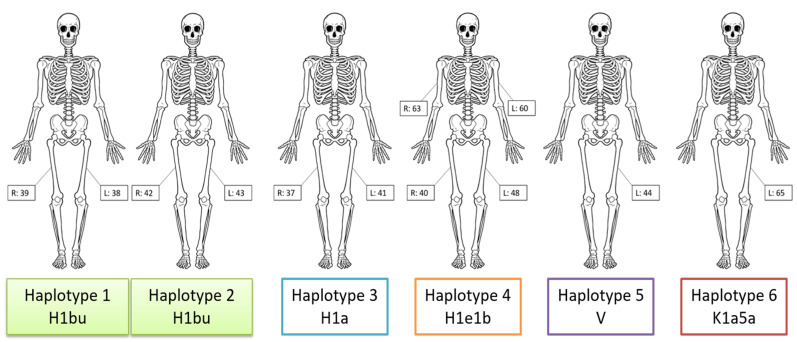
Reassociated skeletal elements based on mitogenome haplotype. Six unique haplotypes were identified, which allowed the skeletal remains to be grouped into a minimum of six individuals. Mitochondrial haplogroups are indicated. R = right and L = left skeletal element.

Six unique mitogenome haplotypes belonging to five separate haplogroups (H1a, H1bu, H1e1b, V, and K1a5a) were identified amongst the 12 samples with reported sequences (Table 2). Based on the uniqueness of the haplotypes observed, skeletal elements could be reassociated (Figure 3), indicating a minimum of six individuals. The two individuals belonging to haplogroup H1bu, represented by samples 38/39 and 42/43, had similar but distinct haplotypes when considering heteroplasmy [19]. Both individuals shared a point heteroplasmy (PHP) at np 13327 (13327R), although variant proportions differed. Samples 38 and 39 yielded ~70% G, whereas samples 42 and 43 had ~83% G. The rCRS has an adenine (A) at np 13327, and the 13327G variant is not diagnostic for haplogroup H1bu. Therefore, the 13327R is a heteroplasmic private polymorphism shared by the two individuals at different variant proportions. In addition to the shared heteroplasmy, an instance of differentiating heteroplasmy was observed between the two H1bu haplotypes. Samples 38 and 39 produced a haplotype with a heteroplasmic insertion at np 12337, 12337.1c, with the lower case “c” indicating the extended IUPAC nomenclature denoting a C/gap heteroplasmy [20]. Approximately 35% of the molecules sequenced from samples 38 and 39 exhibited a cytosine insertion at np 12337, while the remaining molecules had no insertion. The 12337.1 C insertion was absent from the haplotype observed in samples 42 and 43 (0% frequency). Other than the shared and differentiating heteroplasmies at nps 13327 and 12337, respectively, the two H1bu haplotypes were identical. 

### 3.2. Autosomal STR and SNP Sequencing 

Based on the mtDNA sequencing results, autosomal DNA testing was performed on DNA extracts from samples 38 and 43 as well as the buccal swab from Fides Kozulić. Additionally, autosomal STR and SNP data were generated for sample 40 as an unrelated control sample. The results of STR and SNP sequencing from the bone samples are shown in Tables S3–S6. A summary of the locus recovery rate for each sample is shown in Table 3. As expected, no Y-chromosomal markers were obtained from the bone samples or the buccal swab. The X allele at amelogenin was replicated in samples 38, 40 and 43 as well as the buccal swab, thus identifying all four individuals as females. 

Two of three extraction blanks produced data for two SNP loci (rs1005533 and rs576261), and one of these SNP loci (rs1005533) was observed in two of three negative amplification controls. As a result, these two SNP loci (rs1005533 and rs576261) were excluded from all samples for the kinship analysis. None of the control blanks produced replicable STR alleles. The positive control produced the expected profile for 2800M at all loci, including Y-chromosomal STRs and SNPs.

### 3.3. Kinship Analysis

**Figure 4:**
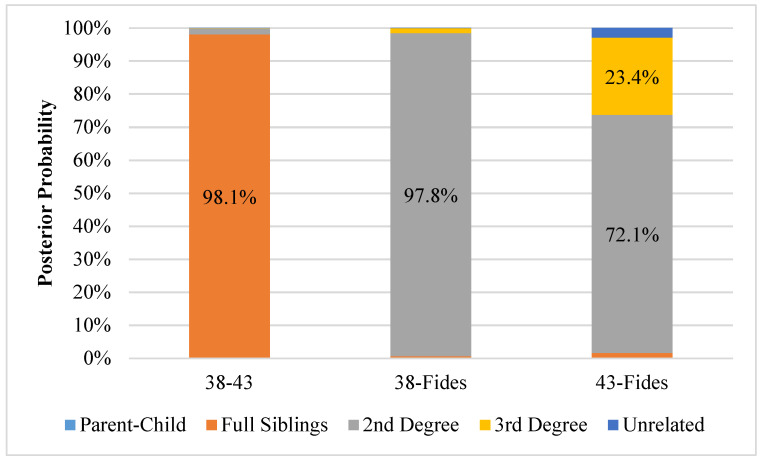
Posterior probability distributions of degrees of relatedness between pairwise comparisons of DNA profiles from samples 38, 43 and the buccal swab (Fides Kozulić)**.** Probabilities greater than 5% are labeled. Sample 40 is not shown because all pairwise comparisons produced likelihood ratios less than one for all degrees of relatedness.

The LRs and posterior probabilities calculated during the blind search in Familias are shown in Table S7. No LR exceeded 1 for any comparison with sample 40, indicating no support for relatedness between the presumed unrelated sample and the other three samples tested. Figure 4 shows the resulting posterior probabilities based on the calculated LRs associated with possible relatedness between samples 38, 43 and Fides. Posterior probabilities >95% were obtained for two relationships assuming equal *a priori* probabilities for each degree of relatedness. Using 39 overlapping loci between samples 38 and 43, a full sibling relationship produced a posterior probability of 98.1% when considering the degrees of relatedness tested (i.e., parent–child, full siblings, 2nd degree, 3rd degree and unrelated). In fact, the LR exceeded 93,000 for a full sibling relationship versus sample 38 and 43 being unrelated. Using the 87 overlapping loci between samples 38 and Fides, an LR of 146,482 was calculated for this 2nd-degree relationship (e.g., aunt-niece) versus the two individuals being unrelated. When comparing the other relationships (including unrelated) with equal priors, the 2nd-degree relationship produced a posterior probability of 97.8%. The kinship analysis for sample 43 and Fides utilized 40 loci, but it resulted in lower LRs overall (maximum of 24) and posterior probabilities of 72.1% and 23.4% for 2nd and 3rd degrees of relatedness, respectively. Posterior probabilities were less than 5% for all other relationships tested between samples 38, 43 and Fides. 

**Figure 5:**
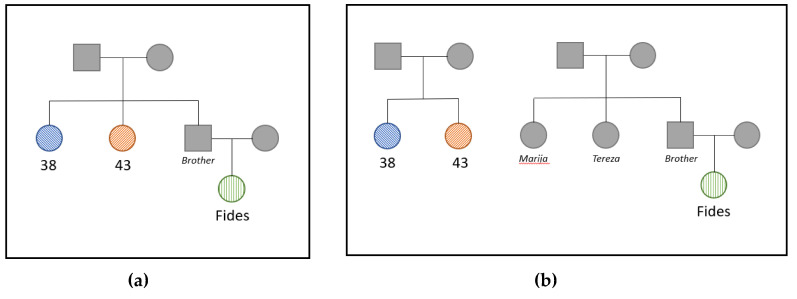
(**a**,**b**) Hypothetical pedigree scenarios compared.

Two hypothetical pedigrees were generated based on case context and mitogenome sequencing. The first pedigree represents the Kozulić family with the presumed full sibling relationship between the two bone samples with a shared mtDNA haplotype (barring heteroplasmy) (38 and 43) and their avuncular relationship with Fides Kozulić (Figure 5a). In the second pedigree samples, 38 and 43 are sisters, but the sisters are unrelated to Fides Kozulić (Figure 5b). Since sample 40 did not indicate a genetic relationship with any of the other samples in the blind search, it was excluded from the pedigree analysis. The likelihoods of producing the observed DNA profiles were compared between the two hypothetical pedigrees. The first pedigree indicating the skeletal remains have a 2nd-degree relationship with Fides Kozulić is 574,195-fold more likely than the alternative in which the skeletal remains are sisters but unrelated to Fides. The posterior probability that the skeletal remains are 2nd-degree relatives of Fides Kozulić is 99.9998%. Therefore, the probability that samples 38 and 43 are unrelated to Fides Kozulić is 0.0002%.

## 4. Discussion

Mitogenome sequencing results allowed for long bones to be sorted by individual. The results supported a minimum of six individuals, each having a unique mitogenome haplotype, among the 14 skeletal elements tested for mtDNA. Although each haplotype was unique when heteroplasmy is considered, only two of the haplotypes shared the same haplogroup (H1bu) indicating a shared maternal ancestor. Moreover, the two H1bu haplotypes exhibit shared heteroplasmy of a private polymorphism, which is not unexpected among close maternal relatives [19,21]. This shared heteroplasmy was observed at two different proportions in the two individual sets of skeletal remains. It is likely that this shared heteroplasmy shifted in proportion from mother to each offspring during the germline bottleneck that occurs in oogenesis [22]. Also of note, the two H1bu haplotypes had differentiating heteroplasmy, as only one of the haplotypes included a heteroplasmic insertion in the mtDNA coding region. It is possible that the differentiating heteroplasmy arose as a germline mutation in one of the sisters, but confirmation would require knowledge of the mother’s haplotype and that is unknown. In addition, given the distinct combination of heteroplasmic signals, if a direct reference from either of the two sisters became available, mtDNA analysis would presumably be able to differentiate Marija from Tereza.

Once the maternal relatives were identified amongst the 14 bone samples tested, autosomal DNA analysis was performed. Only partial STR and SNP profiles were obtained from the bone samples of the two suspected maternal relatives, which is expected from historical remains (e.g., [23,24,25]). Despite the poor DNA quality, especially in sample 43, kinship analysis was possible. The pairwise search indicated that the most likely relationship between the two suspected sisters was a full sibling relationship, which is consistent with historical records. Secondarily, the pedigree analysis provided very strong support for the expected relationship between the skeletal remains and Fides Kozulić, the known paternal niece of Sisters Marija and Tereza. The pairwise kinship analysis also confirmed that sample 40, which was not a suspected maternal relative of the other individuals since a different mitochondrial haplogroup (H1e1b) was observed, was unrelated to Fides Kozulić (as well as samples 38 and 43). This unrelated control bone sample was included in the study to demonstrate that a false-positive result, perhaps occurring due to bias resulting from the age, location, and historical context of the tomb in combination with the allele frequency data obtained from living individuals of European ancestry, is unlikely.

Although the probability that the two sets of remains belong to Sister Marija and Tereza is high, it is impossible to discern which sister is Marija using DNA due to a lack of direct references. One remaining distinction between the sisters may be their height, but this information can only be gleaned from pictures. Judging from the photograph of the monastery and using the height of wall stones as a guide, the stature was estimated to be 166 cm for Marija and 176 cm for Tereza. From the femoral bone measurements, samples 42 and 43 belong to a person estimated at 165.7 ± 3.72 cm, and samples 38 and 39 to a person whose height was estimated at 163.0 ± 3.72 cm. However, given the standard error of measurement in stature estimation, combined with stature change related to aging, as well as the unreliability of the sisters’ height estimation from a historical photograph, it is not possible to determine which remains belong to Sister Marija anthropometrically. Although modern science can tell us that the remains of the Kozulić sisters have been found in the tomb of the Society of Sisters of the Sacred Heart of Jesus, DNA cannot tell them apart. Nonetheless, this information would allow the church to move forward with the beatification of Sister Marija.

## Figures and Tables

**Table 1:** Mitogenome sequencing results obtained from the 14 skeletal samples. The sample identification (ID) (e.g., 37.1) indicates the bone sample (37) with the DNA extract number shown after the decimal (.1 for first DNA extract). rCRS = revised Cambridge Reference Sequence [6].

Sample ID	Reads	% Reads Mapped to rCRS	Reads Mapped to rCRS	Unique Reads Mapped to rCRS	Mean Mapped Read Length	Average Read Death	Bases ≥ 10X
37.1	673,054	10.72%	72,174	68,242	94.88	183.1	16,569
37.2	798,954	8.91%	71,213	66,379	97.33	182.7	16,569
38.1	9,408,738	65.47%	6,159,871	2,711,409	120.98	9557.6	16,569
38.2	8,937,744	45.81%	4,094,273	2,163,767	134.69	8578.4	16,569
38.3	18,372,080	2.10%	386,546	306,340	95.39	827.2	16,569
39.1	1,865,914	45.69%	852,586	713,852	130.54	2717.3	16,569
39.2	8,216,798	16.20%	1,331,479	1,024,117	130.95	3872	16,569
40.1	8,734,042	76.51%	6,682,424	4,216,710	111.65	13,641.1	16,569
40.2	4,369,510	72.15%	3,152,582	2,214,763	107.61	6846.9	16,569
40.3	6,759,524	55.25%	3,734,350	2,370,261	96.16	6689.7	16,569
41.1	3,305,366	48.98%	1,618,905	1,416,205	119.74	4790.5	16,569
41.2	2,818,352	49.54%	1,396,161	1,238,362	119.16	4147.6	16,569
42.1	5,451,990	81.18%	4,425,748	3,065,788	107.65	9555.1	16,569
42.2	9,205,980	80.18%	7,381,722	4,674,287	105.68	14,355.1	16,569
43.1	2,596,506	43.95%	1,141,189	949,031	98.46	2643.8	16,569
43.2	2,856,978	34.62%	989,133	832,220	96.86	2285.1	16,569
43.3	14,864,996	70.57%	10,489,877	4,013,492	86.22	10,229.2	16,569
44.1	1,751,348	1.02%	17,928	17,525	102.58	48	16,569
44.2	2,184,922	1.84%	40,259	39,103	99.71	104.4	16,569
45.1	1,718,530	1.95%	33,482	30,812	117.08	102.2	16,569
45.2	116,968	1.67%	1953	1877	87.91	4.6	1376
46.1	1,799,746	0.01%	195	191	102.53	0.2	0
46.2	891,934	0.03%	226	217	104.73	0.3	0
48.1	6,485,124	76.42%	4,955,891	3,167,070	123.58	11,315.9	16,569
48.2	11,188,962	72.89%	8,155,496	4,684,708	126.54	17,251.5	16,569
60.1	2,339,920	40.06%	937,401	709,827	114.12	2367.6	16,569
60.2	1,692,924	41.48%	702,247	533,893	117.25	1823.8	16,569
63.1	873,150	12.30%	107,368	100,670	116.93	332.2	16,569
63.2	940,844	12.53%	117,848	109,408	119.97	371.8	16,569
65.1	815,424	46.94%	382,728	341,506	115.76	1148.5	16,569
65.2	222,180	5.62%	12,479	12,081	139.45	47.6	16,569

**Table 2:** The six mitogenome haplotypes observed amongst the 12 successfully sequenced skeletal samples. Corresponding haplogroups are indicated. Length heteroplasmy is indicated by “.1” with a lower case letter designating the inserted base (e.g., 12337.1c). Insertions are indicated by “.1” with a capital letter designating the inserted base.

Sample(s)	Haplogroup	Haplotype
38, 39	H1bu	263G 309.1C 315.1C 750G 1438G 3010A 4769G 5558G 8860G 12337.1c 13327R 15326G 16519C
42, 43	H1bu	263G 309.1C 315.1C 750G 1438G 3010A 4769G 5558G 8860G 13327R 15326G 16519C
37, 41	H1a	73G 263G 309.1C 315.1C 750G 1438G 3010A 4769G 8860G 15326G 16162G 16519C
40, 48, 60, 63	H1e1b	263G 309.1C 315.1C 453C 750G 1438G 3010A 4769G 5460A 8512G 8860G 10274C 15326G 16519C
44	V	72C 263G 315.1C 750G *1327R* 1438G 2706G 4580A 4769G 7028T 8860G 12408C 15326G 15904T 16298C
65	K1a5a	73G 263G 315.1C 497T 524.1A 524.2C 750G 1189C 1438G 1811G 2706G 3480G 4640T 4769G 7028T 8860G 9055A 9647C 9698C 10398G 10550G 11017C 11299C 11467G 11719A 12308G 12372A 14167T 14766T 14798C 15326G 16093Y 16129A 16224C 16311C 16362C 16519C

**Table 3:** The number of autosomal short tandem repeat (STR) and single nucleotide polymorphism (SNP) loci produced from three skeletal samples (38, 40 and 43) and Fides Kozulić’s buccal swab. Except for sample 40 (the bone control), each allele was replicated in two independent DNA extracts. The alleles produced from sample 40 were replicated in a second amplification event from the same DNA extract.

Sample	AuSTRs (*n* = 29)	SNPs (*n* = 90)	Total Loci (*n* = 119)
38	22 (76%)	67 (74%)	89 (75%)
40	29 (100%)	71 (79%)	100 (84%)
43	4 (14%)	38 (42%)	42 (35%)
Buccal	27 (93%)	85 (94%)	112 (94%)

## Supplementary Materials

The following are available online at https://www.mdpi.com/2073-4425/11/8/938/s1, Figure S1: The tomb of the nuns, Table S1: Samples tested for DNA, Table S2: Mitogenome sequencing results obtained from the control blanks, Table S3: Sample 38 STR and SNP results for each DNA extract (e.g., 38.2 or 38.3) and amplification event (e.g., 38.5a or 38.5b), Table S4: Sample 40 STR and SNP results for each amplification event (40.3a or 40.3b), Table S5: Sample 43 STR and SNP results for each DNA extract (e.g., 43.2 or 43.3) and amplification event (e.g., 43.5a or 43.5b), Table S6: Fides Kozulic buccal sample STR and SNP results for each DNA extract (e.g., Buccal 1 or Buccal 2), Table S7: Likelihood ratio (versus unrelated) and posterior probability of various degrees of relatedness for pairwise comparisons of all samples.

## References

[1] Mlakić D. (2017). Mother Marija Krucifiksa Kozulić in Risika (In Croatian). Družba Sestara Presvetog Srca Isusova: Postulatura Službenice Božje Majke Marije Krucifikse Kozuli.

[2] Daniels-Higginbotham J., Gorden E.M., Farmer S.K., Spatola B., Damann F., Bellantoni N., Gagnon K.S., de la Puente M., Xavier C., Walsh S. (2019). DNA Testing Reveals the Putative Identity of JB55, a 19th Century Vampire Buried in Griswold, Connecticut. Genes.

[3] Marshall C., Sturk-Andreaggi K., Daniels-Higginbotham J., Oliver R.S., Barritt-Ross S., McMahon T.P. (2017). Performance Evaluation of a Mitogenome Capture and Illumina Sequencing Protocol using Non-Probative, Case-Type Skeletal Samples: Implications for the use of a Positive Control in a Next-Generation Sequencing Procedure. Forensic Sci. Int. Genet..

[4] Gorden E.M., Sturk-Andreaggi K., Marshall C. (2018). Repair of DNA Damage Caused by Cytosine Deamination in Mitochondrial DNA of Forensic Case Samples. Forensic Sci. Int. Genet..

[5] Gallimore J.M., McElhoe J.A., Holland M.M. (2018). Assessing Heteroplasmic Variant Drift in the mtDNA Control Region of Human Hairs using an MPS Approach. Forensic Sci. Int. Genet..

[6] Andrews R.M., Kubacka I., Chinnery P.F., Lightowlers R.N., Turnbull D.M., Howell N. (1999). Reanalysis and Revision of the Cambridge Reference Sequence for Human Mitochondrial DNA. Nat. Genet..

[7] Sturk-Andreaggi K., Peck M.A., Boysen C., Dekker P., McMahon T.P., Marshall C.K. (2017). AQME: A Forensic Mitochondrial DNA Analysis Tool for Next-Generation Sequencing Data. Forensic Sci. Int. Genet..

[8] Holland M.M., Pack E.D., McElhoe J.A. (2017). Evaluation of GeneMarker^®^ HTS for Improved Alignment of mtDNA MPS Data, Haplotype Determination, and Heteroplasmy Assessment. Forensic Sci. Int. Genet..

[9] Gill P., Whitaker J., Flaxman C., Brown N., Buckleton J. (2000). An Investigation of the Rigor of Interpretation Rules for STRs Derived from Less than 100 Pg of DNA. Forensic Sci. Int..

[10] Whitaker J.P., Cotton E.A., Gill P. (2001). A Comparison of the Characteristics of Profiles Produced with the AMPFlSTR SGM Plus Multiplex System for both Standard and Low Copy Number (LCN) STR DNA Analysis. Forensic Sci. Int..

[11] Egeland T., Mostad P.F., Mevag B., Stenersen M. (2000). Beyond Traditional Paternity and Identification Cases. Selecting the most Probable Pedigree. Forensic Sci. Int..

[12] Kling D., Tillmar A.O., Egeland T. (2014). Familias 3–Extensions and New Functionality. Forensic Sci. Int. Genet..

[13] Durbin R.M., Abecasis G.R., Altshuler D.L., Auton A., Brooks L.D., Durbin R.M., Gibbs R.A., Hurles M.E., McVean G.A., 1000 Genomes Project Consortium (2010). A Map of Human Genome Variation from Population-Scale Sequencing. Nature.

[14] 1000 Genomes Project Consortium (2015). A Global Reference for Human Genetic Variation. Nature.

[15] Phan L., Jin Y., Zhang H., Qiang W., Shekhtman E., Shao D., Revoe D., Villamarin R., Ivanchenko E., Kimura M. ALFA: Allele Frequency Aggregator. National Center for Biotechnology Information, U.S. National Library of Medicine. www.ncbi.nlm.nih.gov/snp/docs/gsr/alfa/.

[16] Steffen C.R., Coble M.D., Gettings K.B., Vallone P.M. (2017). Corrigendum to ‘;U.S. Population Data for 29 Autosomal STR Loci’ [Forensic Sci. Int. Genet. 7 (2013) e82-e83]. Forensic Sci. Int. Genet..

[17] Ruitberg C.M., Reeder D.J., Butler J.M. (2001). STRBase: A Short Tandem Repeat DNA Database for the Human Identity Testing Community. Nucleic Acids Res..

[18] Gjertson D.W., Brenner C.H., Baur M.P., Carracedo A., Guidet F., Luque J.A., Lessig R., Mayr W.R., Pascali V.L., Prinz M. (2007). ISFG: Recommendations on Biostatistics in Paternity Testing. Forensic Sci. Int. Genet..

[19] Holland M., Makova K., McElhoe J. (2018). Deep-Coverage MPS Analysis of Heteroplasmic Variants within the mtGenome Allows for Frequent Differentiation of Maternal Relatives. Genes.

[20] Parson W., Gusmao L., Hares D., Irwin J., Mayr W., Morling N., Pokorak E., Prinz M., Salas A., Schneider P. (2014). DNA Commission of the International Society for Forensic Genetics: Revised and Extended Guidelines for Mitochondrial DNA Typing. Forensic Sci. Int. Genet..

[21] Ma K., Zhao X., Li H., Cao Y., Li W., Ouyang J., Xie L., Liu W. (2018). Massive Parallel Sequencing of Mitochondrial DNA Genomes from Mother-Child Pairs using the Ion Torrent Personal Genome Machine (PGM). Forensic Sci. Int. Genet..

[22] Rebolledo-Jaramillo B., Su M.S., Stoler N., McElhoe J.A., Dickins B., Blankenberg D., Korneliussen T.S., Chiaromonte F., Nielsen R., Holland M.M. (2014). Maternal Age Effect and Severe Germ-Line Bottleneck in the Inheritance of Human Mitochondrial DNA. Proc. Natl. Acad. Sci. USA.

[23] Parsons T.J., Huel R.M., Bajunović Z., Rizvić A. (2019). Large Scale DNA Identification: The ICMP Experience. Forensic Sci. Int. Genet..

[24] Zavala E.I., Rajagopal S., Perry G.H., Kruzic I., Bašić Ž, Parsons T.J., Holland M.M. (2019). Impact of DNA Degradation on Massively Parallel Sequencing-Based Autosomal STR, iiSNP, and Mitochondrial DNA Typing Systems. Int. J. Leg. Med..

[25] Alonso A., Andelinovic S., Martin P., Sutlovic D., Erceg I., Huffine E., de Simon L.F., Albarran C., Definis-Gojanovic M., Fernandez-Rodriguez A. (2001). DNA Typing from Skeletal Remains: Evaluation of Multiplex and Megaplex STR Systems on DNA Isolated from Bone and Teeth Samples. Croat. Med. J..

